# Periparturient alpha-lipoic acid supplementation improves ewe antioxidant status, colostrum quality, and lamb growth

**DOI:** 10.1007/s11250-025-04831-8

**Published:** 2026-01-12

**Authors:** Mohammed Hamed Eldawy, Emad Mohammed Elken, Ismail Abdelraouf Elnady, Hanaa A. E. Asfour, El-Shaimaa N. Mehany, Mahmoud Gamal Rashad, Haney Samir, Hossam R. El-Sherbiny, Nadia Hamdi Fahim

**Affiliations:** 1 Animal Production Department, Faculty of Agriculture, Al-Azhar University, Nasr City, Cairo Egypt; 2 Mastitis and Neonatal Diseases Department, Animal Reproduction Research Institute (ARRI), Agricultural Research Center (ARC), Giza, Egypt; 3 Theriogenology Department, Faculty of Veterinary Medicine, Cairo University, Giza, Egypt; 4 Animal Production Department, Faculty of Agriculture, Cairo University, Giza, Egypt

**Keywords:** Periparturient, Ewe, Alpha lipoic acid, Immunity, Milk quality, Lamb growth

## Abstract

Alpha-lipoic acid (α-LA) is a potent antioxidant with potential to enhance ruminant health and productivity, yet its effects in late-pregnant ewes are not well defined. This study evaluated α-LA supplementation (600 mg/day) from 60 days prepartum to 8 weeks postpartum on blood biochemistry, immunity, colostrum and milk composition, microbiological quality, and lamb performance in Egyptian ewes. Thirty ewes were randomly allocated to control or α-LA groups (*n* = 15). On day one postpartum, α-LA ewes had higher serum albumin (2.87 vs. 2.45 g/dL), globulin (2.76 vs. 2.58 g/dL), total protein (5.64 vs. 5.04 g/dL), and total antioxidant capacity (29.97 vs. 28.19 mM/L; *p* ≤ 0.01). Elevated immunoglobulin A persisted at 4 weeks (80.20 vs. 73.40 ng/mL; *p* = 0.02). Lambs from supplemented ewes showed greater IgA (135.40 vs. 89.00 ng/mL; *p* = 0.04), glutathione peroxidase (29.16 vs. 20.51 U/mL; *p* < 0.05), TAC (56.48 vs. 31.80 mM/L; *p* < 0.01), and higher birth (4.52 vs. 3.84 kg) and weaning weights (7.39 vs. 6.00 kg; *p* < 0.01). Colostrum from α-LA ewes contained more fat (16.75 vs. 7.98%), protein (8.91 vs. 6.66%), and total immunoglobulins (21.47 vs. 5.84; *p* < 0.01), while both colostrum and milk had reduced bacterial counts and prevalence of *Staphylococcus aureus*,* Escherichia coli*,* Klebsiella spp*., and *Bacillus cereus*. In conclusion, periparturient α-LA supplementation improved maternal immunity, antioxidant capacity, colostrum and milk quality, and lamb growth. Further research should determine optimal dosing, assess long-term impacts on milk yield, and explore its integration into mastitis control programs.

## Introduction

Oxidative stress is a popular concern in livestock that impacts health and performance. Alpha-lipoic acid (α-LA) is a natural compound that has a high antioxidant activity, which acts as a coenzyme in mitochondrial energy metabolism. Studies have assessed the potential benefits of α-LA supplementation in various breeds of livestock. In chickens, α-LA supplementation affected the eggshell quality and serum estradiol level, representing improved reproduction in the advanced age of egg-laying life (Dai 2013). Furthermore, α-LA could enhance the body composition in ducks by decreasing fat deposition and increasing muscle growth (El-Rayes 2020). In piglet litters from Duroc and Yorkshire breed sows, α-LA administration could enhance antioxidant defense and growth performance (Li et al. 2022). α-LA supplementation in Holstein cattle increased milk yield and enhanced metabolism, which positively impacted productivity and economics (Zhang et al. 2024). In addition, it improved blood flow to the tests and semen quality in aging bucks, suggesting a potential role in slowing reproductive aging (Abdelnaby et al. 2024).

Sheep research has found that α-LA supplementation can enhance antioxidant enzyme activity, reduce oxidative stress, and impact rumen fermentation processes to increase sheep productivity and health. α-LA has also been supplemented to enhance antioxidant enzyme activity, such as superoxide dismutase and glutathione peroxidase, in sheep and reduce malondialdehyde, which has been employed as a marker of oxidative stress (Yang et al. 2023). α-LA in the dose of 600 mg/kg significantly enhanced antioxidant activity in the rumen by regulating metabolic activity (Yao et al. 2024). Growth efficiency, feed efficiency, sheep daily weight gain of 246 g, and feed conversion ratios were enhanced with supplementation of 80 ppm of α-LA (Robles-Rodríguez et al. 2022). The more concentrated level (120 ppm) caused no significant changes in blood glucose and volatile fatty acid concentrations (Robles et al. 2019), which reflects the intricate interaction between rumen microbiota.

Besides, α-LA changed the rumen microbiota by enhancing cellulolytic bacteria but reducing overall bacteria, enhancing fiber digestion (Huerta-Jiménez et al. 2023). Further, α-LA supplementation improved postmortem lamb muscle color stability through changing types of muscle fibers and antioxidative status (Luo et al. 2022).

The prepartum period in sheep is characterized by profound physiological, metabolic, and immunological adjustments that affect maternal and neonatal outcomes. Excessive metabolism from gestation and parturition in ewes may increase oxidative stress, hepatic postpartum strain, and immune drop, ultimately compromising colostrum quality (Du et al. 2025). Poor immunity and nutrition would negatively impact maternal and neonatal health and survival. Therefore, warranting optimum nutrition during late gestation and early lactation is urgent for ewe and lamb performance (Ji et al. 2023).

Despite extensive evidence of α-LA benefits in poultry, pigs, and cattle, its role in sheep during the periparturient period remains unexplored. No previous study has investigated whether maternal α-LA supplementation can modulate ewe antioxidant status, improve colostrum and milk hygiene, and enhance lamb growth performance. Addressing this gap provides the first evidence for α-LA as a promising nutritional strategy to optimize maternal–neonatal health and productivity in sheep production systems. Therefore, this study aimed to examine the impacts of prepartum α-LA supplementation on ewe antioxidant status, colostrum and milk quality, and lamb growth performance.

## Materials and methods

### Ethical approval

This study is approved and accredited by the Ethical Committee for the Use and Care of Animals at the Faculty of Agriculture, Al-Azhar University (Approval No. AZHU6D625).

### Animals

The ewes of this study belonged to the experimental and research farm of the Faculty of Agriculture, Al-Azhar University, Cairo, Egypt. The geographical coordinates of the farm are 30°03′14″N 31°19′04″E. The study included thirty Egyptian ewes with a body weight of 45–50 ± 0.5 kg and in 90–95 days of gestation based on breeding records in the farm. Ewes were housed in semi-open pens under a temperature of 30–43 °C and a relative humidity of 45–53%. Ewes were fed on a balanced ration following the NRC guidelines. The concentrated feed included 70% TDN, 14% crude protein, 8% fiber, 4% ether extract, and 4% ash. It comprises 51% yellow corn, 24% wheat bran, 12% cottonseed cake, 6% soybean meal, 1.5% ground limestone, 5% common salt, and 0.5% mineral-vitamin premix. Water, minerals, and roughage (alfalfa hay and rice straw) were provided ad libitum for all animals. The ewes were exposed for an hour/day to natural sun rays (4.00–5.00 pm). All animals were healthy and clinically free from diseases.

### Experimental design

The study started in May and lasted until mid-July 2025. Thirty ewes during mid-gestation (about week 10 of the gestation) were enrolled in the current experiment. Ewes weighing 47.5 ± 0.5 kg and 3.55 ± 0.62 years old were randomly assigned to two cohorts based on their body condition score and liveweight. All ewes were of the same age and parity (third parity) and had one litter size, which was confirmed through farm records and ultrasound. Randomization was performed using a simple random number generator to assign animals to either the control or α-LA-supplemented group. Ewes of the control group were provided with the basal diet only, while ewes of the α-LA group were supplied with the basal diet + α-LA (600 mg/ewe/day). At the beginning of the study, the ewes’ average weight was 47.5 ± 0.5 kg, meaning that the daily dose was roughly 600 mg/day for each ewe or 12.63 mg/kg bodyweight. A dose supported by prior research demonstrating antioxidant activity in ruminants at a similar mg/kg of dry matter (Zhang et al. 2021). Making it clear that the supplement is racemic α-Lipoic Acid (a combination of R and S enantiomers), the most prevalent type in animal experiments. The α-LA dose was dissolved in 50 mL of clean water and orally drenched to each ewe once daily using a syringe without a needle.

### Udder size measurement

Udder was measured once weekly throughout the experimental period using a flexible measuring tape (in centimeters). Measurements were taken before the morning milking (7:00 am) to avoid variation due to milk accumulation. Three anatomical parameters were recorded for each ewe according to the method of Bencini and Purvis (1990): udder length; the distance from the base of udder attachment to the lowest point of the teat, udder width; the horizontal distance between the outer margins of the two halves of the udder, and udder depth; the vertical distance from the udder base to the midpoint between the teats. The average of the three measurements was considered the udder size (cm). To account for diurnal variation, milk yield (L/day) was measured by hand milking at weeks 2, 4, 6, and 8 postpartum (7:00 am and 7:00 pm).

### Estimating the body weight of yielded lambs

The study recorded individual lamb weight gain and calculated their weekly weighed until the sixth week, Initial weight (birth weight): was recorded within 12–24 h after birth (kg), Subsequent weights: lambs were weekly weighed until the sixth week (kg), and Average Daily Gain = Final Weight − Initial Weight/No. of days between measurements (kg/day).

### Blood sampling

Blood samples (10 mL, jugular vein) from ewes (at parturition and four weeks postpartum) and (3 mL, jugular vein) from lambs (once at parturition). Samples were collected before the morning feeding at (8:00 am ± 30 min) to account for changes in the day. However, the ewes were not fasting, and centrifuged at 3000×g for 15 min at 4 °C. The plasma was collected and frozen at -20 °C until biochemical analysis.

### Colostrum and whole milk sampling

Ewes involved in the study were of the same age and parity (third parity) and had a single litter size confirmed by farm records and ultrasonography to minimize individual variability. Colostrum samples (25 mL/ewe) were collected at parturition and on day 3 postpartum, while whole milk samples (25 mL/ewe) were obtained biweekly from the second to the eighth week postpartum.

All lambs were allowed to suckle their dams naturally within 1–2 h after parturition to ensure adequate colostrum intake and effective passive transfer of immunity. Lambs’ behavior was continuously monitored to ensure successful suckling.

For experimental consistency and reproducibility, lambs were separated from their dams for about 12 h before sampling to permit full udder distension and complete milk let-down (Bencini and Pulina 1997). Ewes were calmly restrained in a hygienic environment, and all sampling procedures were conducted under aseptic conditions. Personnel involved in sample collection wore sterile disposable gloves and clean laboratory coats, and sterile plastic tubes were used as collection vessels (Middleton et al. 2014). Before sampling, udders and teats were thoroughly washed with warm water, dried using disposable paper towels, and then wiped with 70% ethanol to ensure surface disinfection. The initial milk jets (two or three streams) were discarded to remove potential contaminants from the teat canal (Farooq et al. 2024). Midstream milk was subsequently hand-expressed directly into sterile vials, avoiding contact with the rim or cap.

Immediately after sealing and labeling, samples were placed in insulated containers at approximately − 4 °C during transport, and delivered to the laboratory within four hours or frozen at − 20 °C for long-term storage (Bruckner 2009). The rigorous hygiene protocol applied throughout sample collection minimized the risk of exogenous contamination and controlled for confounders that might affect colostrum and milk composition or microbiological results.

### Blood and serum biochemical analyses

Plasma biochemical and immunological parameters were measured using commercial kits according to the manufacturers’ instructions. All analyses were performed in triplicate to ensure repeatability.

Albumin was determined colorimetrically using bromocresol green reagent (Biodiagnostic Co., Egypt; CAT No. AB1010; sensitivity = 0.2 g/dL; intra-assay CV < 5%) as described by Doumas et al. (1971). Alkaline phosphatase (ALP) activity was assayed using *p*-nitrophenyl phosphate substrate (Biodiagnostic Co., Egypt; CAT No. ALP1010; λ = 405 nm) according to Belfield and Goldberg (1971). Aspartate and alanine aminotransferases (AST and ALT) were analyzed using enzymatic colorimetric kits (Biodiagnostic Co., Egypt; CAT No. AS1061(45), AL1031(45); λ = 505 nm) following Reitman and Frankel (1957). Creatinine was measured colorimetrically (Biodiagnostic Co., Egypt; CAT No. CR1251; λ = 495 nm) as per Henry et al. (1975).

Immunoglobulin A (IgA) concentration was determined using a sheep ELISA kit (Sunlong Biotech Co., China; CAT No. SL00021Sp; sensitivity = 0.1 µg/mL; λ = 450 nm). Total antioxidant capacity (TAC) and glutathione peroxidase (GPx) were assayed colorimetrically using commercial kits (Biodiagnostic Co., Egypt; CAT No. TA2513) following Koracevic et al. (2001) and Paglia and Valentine (1967).

### Colostrum and milk analyses

#### Chemical composition of colostrum and milk samples

A Lactoscan ultrasonic milk analyzer, Bulgaria-25,010, assessed the chemical composition of colostrum and milk samples.

#### Enzyme’s assessment

Alkaline phosphatase (ALP) and glutathione-peroxidase (GPx) in milk were assessed by commercial colorimetric and kinetic assay kits (Biodiagnostic Company, Egypt) using a spectrophotometer (MAPADA instruments UV1800PC) at different wavelengths. The results were expressed as U/L milk.

#### Total Immunoglobulin (Ig) assessment

Total Immunoglobulin (Ig) levels were determined according to Pompermayer et al. (2019). The samples were tested with the zinc sulfate turbidity test (ZST). The TEMIS LiNEAR chemistry analyzer, REF 1,803,050, Montagat, Spain (EU), measured the Ig levels. The standard solution used to interpret results had a turbidity corresponding to approximately 800 mg/dL of Ig.

#### Microbiological analyses

Colostrum and milk samples were inspected under complete aseptic conditions to isolate the different microbial species. A loopful of previously incubated samples at 37 °C for 24 h was sown on blood agar, Mannitol salt agar, MacConkey agar, Eosin methylene blue agar, Edward’s media, and HiCrome Bacillus agar (HiMedia) for bacterial isolation. Sabouraud dextrose agar (HiMedia) was used to isolate yeast. The cultivated media were incubated for 1–2 days at 37 °C. Microbial species were determined by inspecting cultural and morphological features using Gram stain and biochemical identification.

Quantitative enumeration of microorganisms (CFU/mL) was not feasible because several colostrum samples, particularly those collected during night-time lambing, were transported to the microbiology laboratory the following day. These samples were therefore considered non-fresh, and bacterial viability could not be reliably maintained for accurate counting. Accordingly, results were expressed qualitatively as the presence or absence of microorganisms (Quinn et al. 2011).

### Statistical analyses

Data were analyzed using SPSS software (version 25.0; IBM Corp., Armonk, NY, USA). Data normality was tested using the Shapiro–Wilk test (*p* > 0.05), and homogeneity of variances was assessed by Levene’s test. For udder size, milk yield, lamb weight, and blood biochemical parameters, independent samples t-tests were applied. Colostrum and milk composition data were analyzed using repeated measures ANOVA, with treatment (control vs. α-LA) as the between-subject factor and time (sampling point) as the within-subject factor. Bonferroni post-hoc adjustments were performed to correct for multiple comparisons. Effect sizes were calculated as Cohen’s *d* for t-tests and partial eta-squared (η²) for ANOVA. Results are presented as mean ± standard error (SE) along with 95% confidence intervals. Exact *p*-values are reported, and significance was accepted at *p* < 0.05 after adjustment for multiple testing.

## Results

### Blood biochemical and immunological profiles in ewes and their lambs

Table 1 shows that at parturition, α-LA supplementation was significantly associated with higher serum albumin, total protein (*p* < 0.01), globulin, and TAC (*p* = 0.01). Creatinine levels were significantly lower in the α-LA group than in the control group (*p* < 0.01). AST activity was higher in the α-LA group (*p* < 0.01), while ALT activity, IgA, and GPx activity levels were not different among groups (*p* = 0.84, 0.44, and 0.84, respectively). TAC was higher in the α-LA group (*p* = 0.01). At 4 weeks postpartum, the α-LA group had higher albumin, globulin, and total protein (*p* ≤ 0.01) than the control group. The difference in creatinine levels between the two groups was not significant. Also, no significant differences were observed in AST and ALT enzyme activities. The ewes in the α-LA group had higher IgA, TAC, and GPx (*p* < 0.01) than those in the control group.

**Table 1:** Effects of alpha-lipoic acid (α-LA) supplementation on blood parameters of Ewes at parturition and four weeks postpartum

	At parturition			Four weeks postpartum		
	(Mean ± SE)			(Mean ± SE)		
Parameter	Control group	α-LA group	*p*-value	Control group	α-LA group	*p*-value
Albumin (g/dL)	2.45 ± 0.01	2.87 ± 0.10	< 0.01	2.28 ± 0.01	3.15 ± 0.09	< 0.01
Globulin (g/dL)	2.58 ± 0.05	2.76 ± 0.09	0.01	2.54 ± 0.12	3.92 ± 0.18	0.01
Total Protein (g/dL)	5.04 ± 0.06	5.64 ± 0.13	< 0.01	4.86 ± 0.13	5.64 ± 0.17	< 0.01
Creatinine (mg/dL)	3.06 ± 0.03	2.94 ± 0.12	< 0.01	2.81 ± 0.16	3.52 ± 0.16	0.23
Aspartate Aminotransferase (AST, U/L)	23.50 ± 1.30	30.10 ± 1.97	< 0.01	27.80 ± 1.35	28.10 ± 1.72	0.38
Alanine Aminotransferase (ALT, U/L)	51.40 ± 1.88	73.60 ± 1.75	0.84	64.50 ± 1.59	87.80 ± 1.75	0.68
Immunoglobulin A (IgA, ng/mL)	74.20 ± 2.43	80.60 ± 2.56	0.44	73.40 ± 1.85	80.20 ± 1.35	0.02
Glutathione Peroxidase (GPx, U/mL)	17.59 ± 1.73	19.17 ± 1.63	0.84	21.94 ± 3.05	25.65 ± 2.02	< 0.01
Total Antioxidant Capacity (TAC, mM/L)	28.19 ± 1.96	29.97 ± 1.19	0.01	21.55 ± 3.13	32.96 ± 4.97	< 0.01

Statistical significance set at *p* < 0.05

**Fig. 1:**
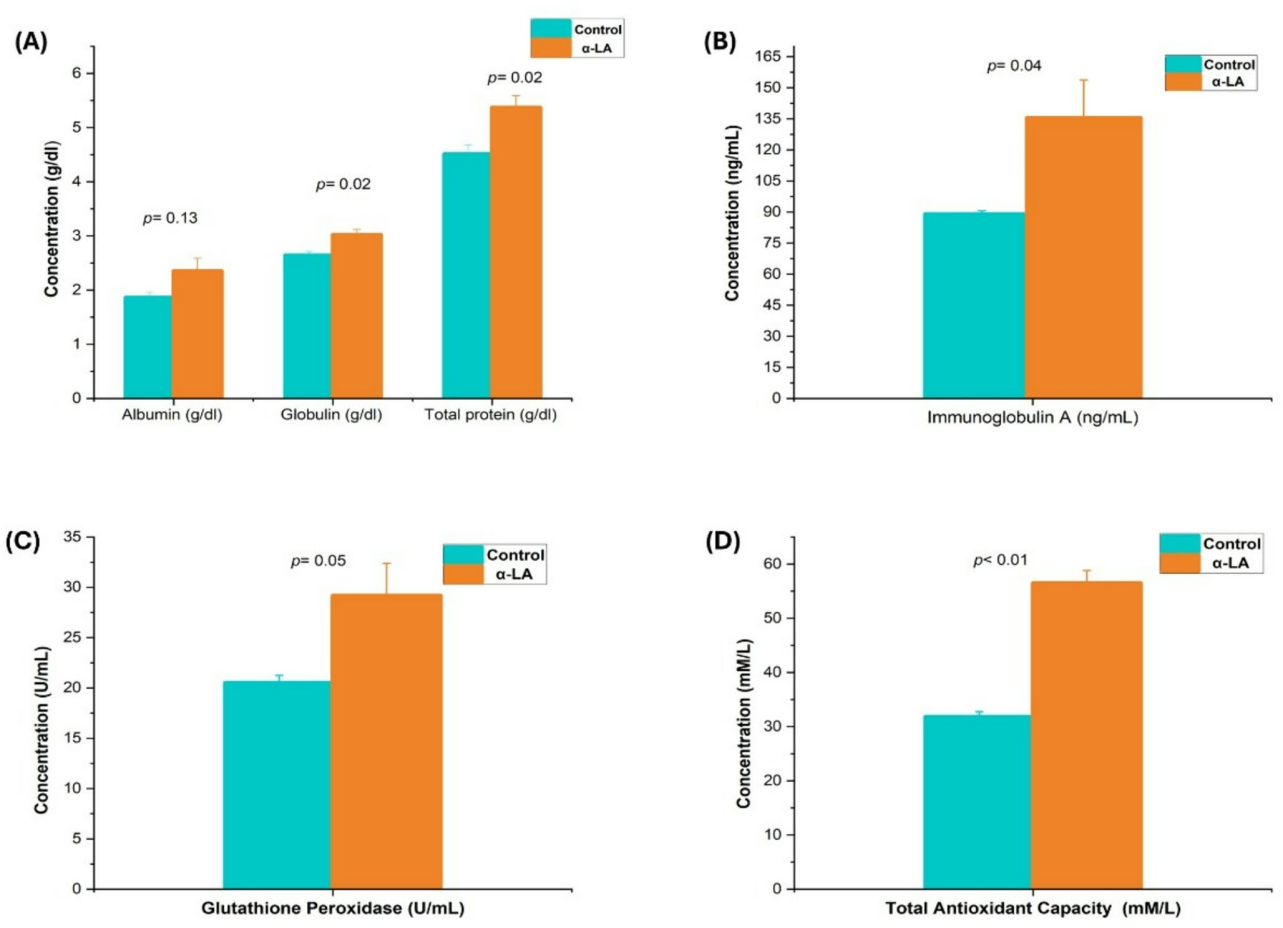
Blood biochemical parameters in lambs from control and alpha-lipoic acid (α-LA) supplemented ewes’ groups; (*p* < 0.05). (**A**) albumin, globulin, total protein, (**B**) immunoglobulin A, (**C**) glutathione peroxidase, (**D**) total antioxidant capacity. All data were expressed as mean ± standard error

Figure 1 shows that lambs from ewes of the α-LA group had higher globulin, total protein (*p* = 0.02), IgA (*p* = 0.04), GPx (*p* < 0.05), and TAC (*p* < 0.01) compared to lambs from the control group (Fig. 1A, B, C; *p* < 0.05).

### Lactational performance of α-LA-supplemented ewes

#### Milk production

**Fig. 2:**
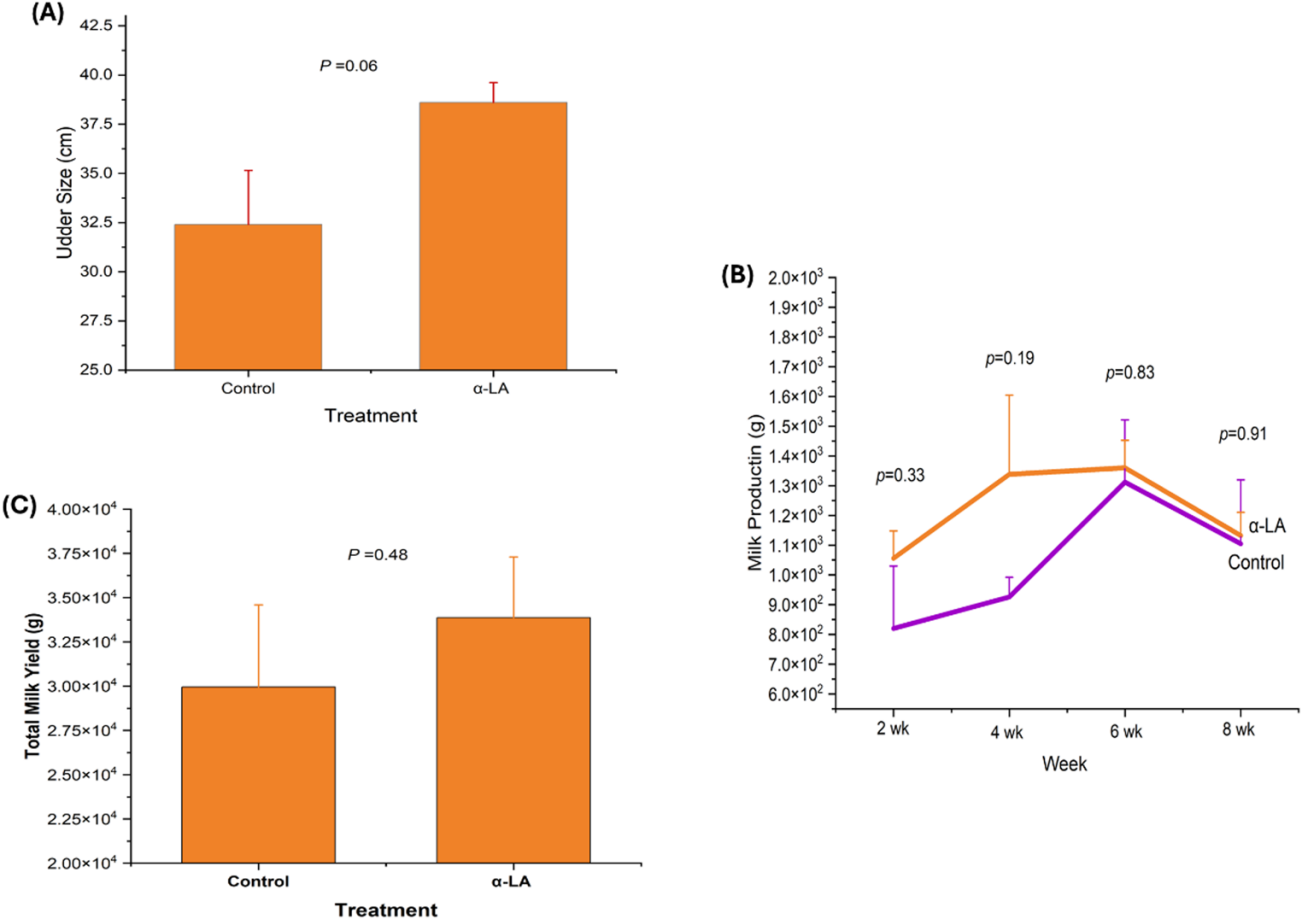
Udder size and milk production in ewes from control and alpha-lipoic acid (α-LA) groups; (*p* = NS). (**A**) Udder size, (**B**) milk production, (**C**) total milk yield. All data were expressed as mean ± standard error

Figure 2 shows that the α-LA-supplemented ewes had a larger mean udder size than the control ewes; however, this was not significantly different (Fig. 2A; *p* = 0.06). Milk yield increased in the α-LA group from week 2 to 8, but the differences were not significant (Fig. 2B and C; *p* = 0.33–0.91).

#### Colostrum composition

Table 2 shows that fat content was higher in the α-LA group at parturition and three days postpartum (*p* < 0.01). Likewise, solid-not-fat (SNF), total immunoglobulin content, and the activity of GPx and ALP were higher in the α-LA group at both time points (*p* = 0.02-< 0.01). In contrast, protein and lactose content were significantly greater at parturition (*p* < 0.01) but not at day three (*p* = 0.07, 0.18).

**Table 2:** Colostrum composition in ewes at parturition and three days postpartum in control and alpha-lipoic acid (α-LA) groups

Parameter	At parturition			At three days postpartum		
	Control group	α-LA group	*p*-value	Control group	α-LA group	*p*-value
Fat (%)	7.98 ± 0.79	16.75 ± 1.12	< 0.01	7.16 ± 0.79	12.75 ± 0.95	< 0.01
Protein (%)	6.66 ± 0.34	8.91 ± 0.49	< 0.01	6.23 ± 0.15	7.48 ± 0.65	0.07
Lactose (%)	5.97 ± 0.16	7.55 ± 0.39	< 0.01	5.94 ± 0.18	6.46 ± 0.32	0.18
Solids-Not-Fat (SNF, %)	12.32 ± 0.52	16.33 ± 0.91	< 0.01	10.94 ± 0.36	14.82 ± 0.80	< 0.01
Ash %	0.99 ± 0.03	1.27 ± 0.08	< 0.01	0.92 ± 0.03	1.10 ± 0.05	< 0.01
Total Immunoglobulin (g/dl)	5.84 ± 0.48	21.47 ± 1.08	< 0.01	4.78 ± 0.45	19.36 ± 0.75	< 0.01
Glutathione Peroxidase (GPx u/l)	2.49 ± 0.20	5.05 ± 0.67	< 0.01	1.93 ± 0.26	3.71 ± 0.54	0.04
Alkaline Phosphatase (ALP u/l)	206.68 ± 30.66	404.56 ± 44.75	< 0.01	180.02 ± 27.79	278.13 ± 26.57	0.02

Statistical significance set at *p* < 0.05

#### Whole milk composition

**Fig. 3:**
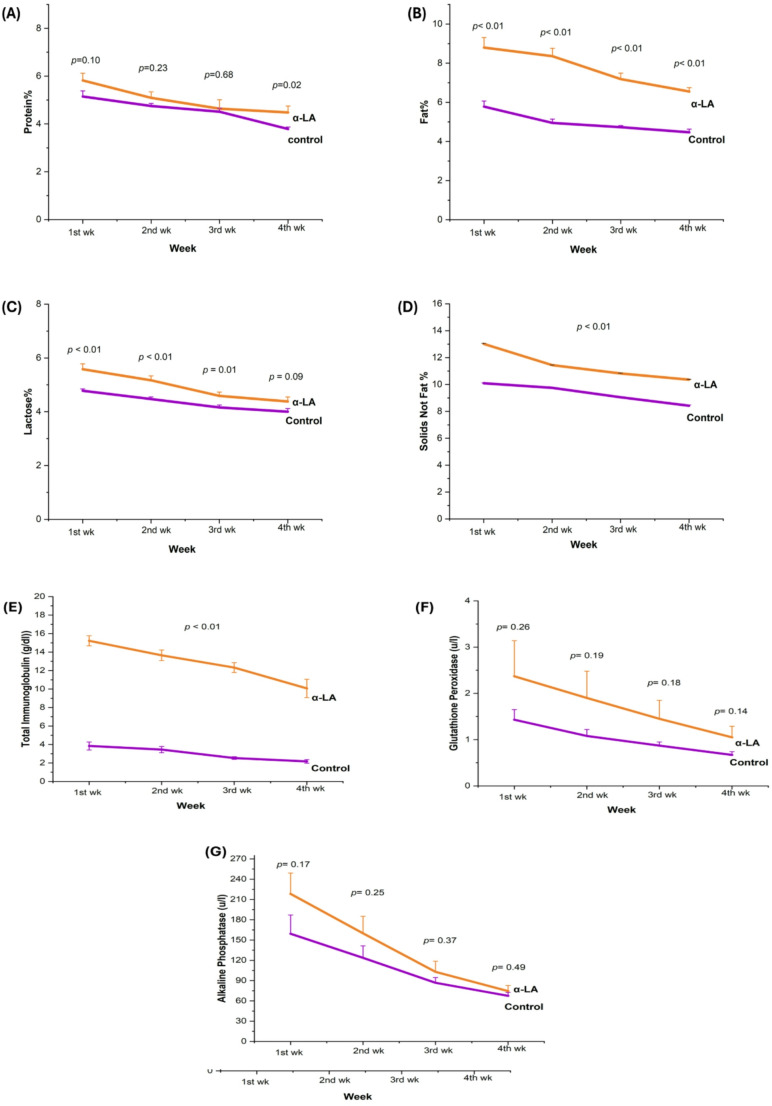
Whole milk composition in ewes over 4 weeks: Control vs. alpha-lipoic acid (α-LA) supplemented groups; (*p* < 0.05). (**A**) protein, (**B**) fat, (**C**) lactose, (**D**) solids not fat, (**E**) immunoglobulin A, (**F**) glutathione peroxidase, (**G**) alkaline Phosphatase. All data were expressed as mean ± standard error

The fat and immunoglobulin levels were significantly higher early in the study (Fig. 3B, E; *p* < 0.01). Lactose was elevated considerably in the α-LA group during weeks one to three (*p* < 0.01), but not in week four (Fig. 3C; *p* = 0.09). Elevated SNF levels (*p* < 0.01) persisted through most weeks (Fig. 3D). Trends toward higher GPx and ALP activities were observed but did not reach statistical significance (Fig. 3F, G; *p* > 0.05).

#### The microbial profile of colostrum and whole milk samples

The results of the microbial analysis of colostrum samples are presented in Table 3. Control colostrum samples showed a high bacterial load, with several species detected up to the third day postpartum. In contrast, α-LA supplementation resulted in no detectable microorganisms in the colostrum. Table 4 shows that, over lactation weeks, microbial analysis showed that control milk had higher content of various bacteria. In contrast, α-LA supplementation significantly reduced microbial presence, with no detectable microorganisms from week two onward and complete microbial absence by weeks three and four.

**Table 3:** Microbial profile in colostrum at parturition and three days postpartum in control and α-lipoic acid (α-LA) supplemented groups

Isolated microorganism	At parturition		At three days postpartum	
	Control	α-LA	Control	α-LA
*Staphylococcus aureus*	50%	–	50%	–
Coagulase-negative *Staphylococci* (CNS)	25%	–	50%	25%
*Escherichia coli*	25%	–	25%	–
*Klebsiella* spp.	–	–	–	–
Environmental *Streptococcus* spp.	25%	–	100%	50%
*Bacillus cereus*	50%	–	–	–
Other *Bacillus* spp. (*B. cereus*)	50%	–	50%	–
Yeast spp.	–	–	–	–

**Table 4:** Microbial profile in whole milk over 4 weeks in control and α-lipoic acid (α-LA) supplemented groups

Isolated Microorganism	1st Week			2nd Week			3rd Week			4th Week	
	Control	α-LA		Control	α-LA		Control	α-LA		Control	α-LA
*Staphylococcus aureus*	**–**	**–**		–	–		–	–		–	–
Coagulase-negative *Staphylococci* (CNS)	100%	–		100%	–		100%	–		100%	–
*Escherichia coli*	100%	50%		–	–		–	–		–	–
*Klebsiella* spp.	–	–		–	–		100%	–		100%	–
Environmental *Streptococcus* spp.	100%	50%		–	–		–	–		100%	–
*Bacillus cereus*	50%	–		100%	–		100%	–		100%	–
Other *Bacillus* spp. (*B. cereus*)	100%	–		–	–		–	–		–	–
Yeast spp.	50%	–		–	–		–	–		–	–

### Growth performance of lambs from α-LA-supplemented ewes

**Fig. 4:**
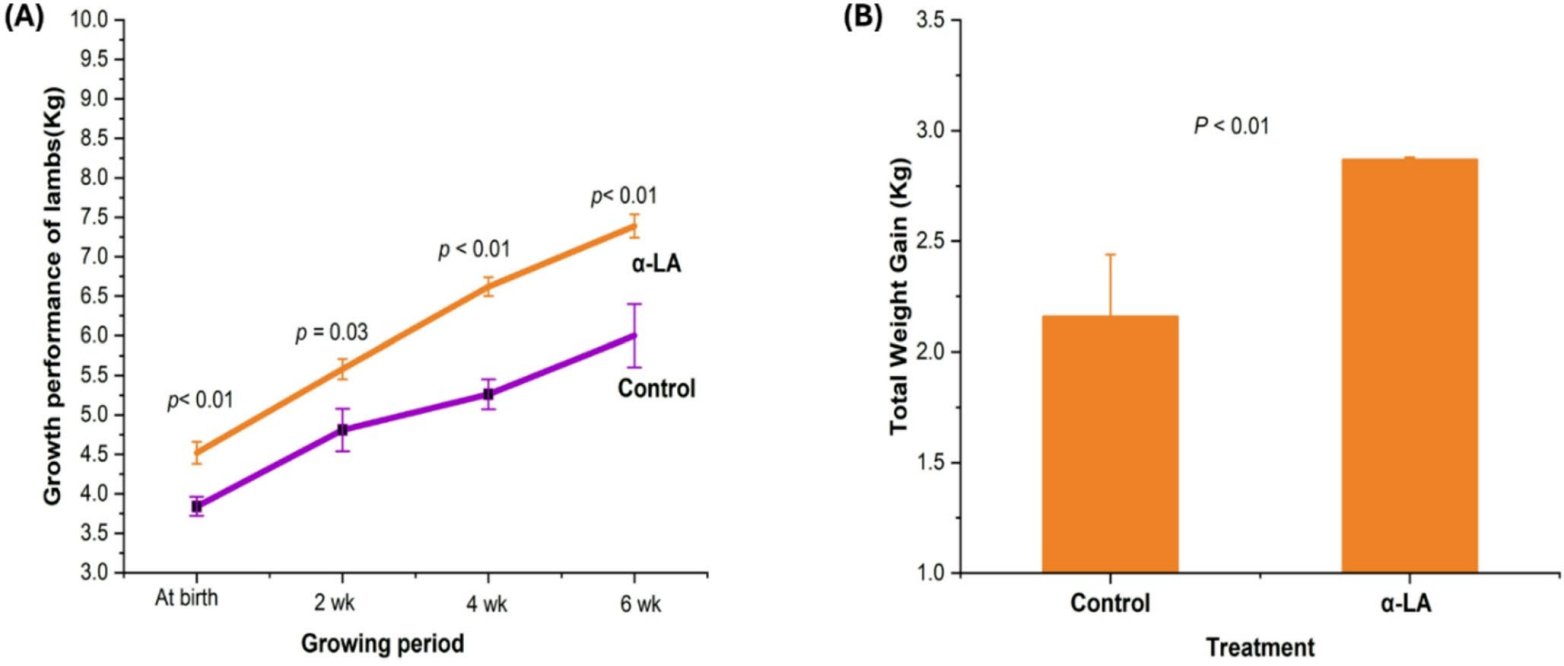
Body weight and weight gain of lambs from control and alpha-lipoic acid (α-LA) supplemented ewes’ groups; (*p* < 0.05). (**A**) growth performance, (**B**) total weight gain. All data were expressed as mean ± standard error

Figure 4 shows that lambs from the α-LA-supplemented ewes showed significantly higher body weights than those from control ewes at birth, 2 weeks, 4 weeks, and weaning (all *p* ≤ 0.03), with a greater overall weight gain by weaning (2.87 kg vs. 2.16 kg) (Fig. 4A, B; *p* < 0.01).

## Discussion

### Maternal health and antioxidant status

The present study demonstrates that α-LA supplementation in prepartum ewes favorably modulated maternal serum protein profiles and antioxidant status, which is practical for neonatal and maternal health. α-LA-treated ewes at parturition had higher albumin, globulin, total protein, and TAC levels, suggesting improved nutritional and oxidative stability. Elevated levels of albumin and globulin indicate improved liver function and immune modulation. Albumin, a major plasma protein, is essential for maintaining oncotic pressure and transporting various internal molecules (Wada et al. 2016). In contrast, globulins, in the form of immunoglobulins, are crucial for immune defense (Ward et al. 2022). Increased albumin in ewes may also facilitate greater transfer of immunoglobulins to lambs, enhancing passive immunity, which is critical for neonatal survival (Tóthová et al. 2023). Conversely, low albumin levels are usually associated with pregnancy toxemia, as the lack of available energy jeopardizes the health of both the mother and the fetus (Khames et al. 2023), highlighting the need for proper nutritional management during pregnancy (Ji et al. 2023).

Contrary to expectations, creatinine was significantly decreased in the α-LA group at the time of parturition, but this effect did not persist after parturition. Lower creatinine levels may reflect improved renal function (Chen et al. 2023) or reduced muscle catabolism during the energetically taxing parturient period (Thongprayoon et al. 2016). This observation warrants confirmation in subsequent studies because postpartum values did not differ significantly.

Another notable observation was the slight elevation of AST activity in α-LA-supplemented ewes at parturition, while ALT levels remained unchanged, and creatinine concentration was significantly decreased. All values were within the normal physiological ranges for healthy ewes: AST 60–280 U/L (Kaneko et al. 2008), ALT 15–60 U/L (Tóthová et al. 2023), and creatinine 0.9–2.0 mg/dL (Khames et al. 2023). The increase in AST, in the absence of ALT elevation, likely reflects normal metabolic or muscular adaptation rather than hepatocellular injury, consistent with previous reports in ruminants linking moderate AST increases without ALT changes to enhanced energy metabolism and mitochondrial activity (Nascimento et al. 2015; Robles et al. 2019; Zhang et al. 2024). The decrease in creatinine at parturition, still within physiological limits, may indicate improved renal function and reduced muscle catabolism during the periparturient period (Chen et al. 2023). Although CK and bilirubin were not measured, the overall biochemical and clinical profile supports normal hepatic and renal function, confirming these changes were adaptive and physiological rather than pathological. Together, these outcomes emphasize α-LA’s ability to enhance maternal antioxidant and protein status, supporting metabolic resilience and neonatal immunity during physiological stress, as previously reported by Yang et al. (2023) and Li et al. (2024).

Besides, elevated IgA in the α-LA group also supports the postulation that α-LA boosts mucosal immunity, possibly through reduction of oxidative stress and an increase in immune cell function. Elevated IgA is especially beneficial during parturition, when immune protection is usually impaired.

The increase in the activities of TAC and GPx confirms that α-LA supplementation improves the antioxidant defense system (Yang et al. 2023). The improvement enables ewes to withstand the oxidative stress involved in parturition and early lactation (Yao et al. 2024). Mechanistically, α-LA has been demonstrated to neutralize reactive oxygen species while recycling other antioxidants such as vitamin C and glutathione (Superti and Russo 2024). Because of the enzyme’s pivotal function in peroxide detoxification, these activities are likely responsible for this study’s enhanced GPx activity.

### Neonatal immunity and antioxidant transfer

Lambs of α-LA-supplemented ewes had superior globulin and total protein concentrations alongside increased IgA levels. This indicates improved passive immunity transfer, likely resulting from enhanced colostral quality attributable to maternal supplementation. Yilmaz and Kasikci (2013) stated that improved colostrum directly translates into higher serum immunoglobulin levels in neonates, reinforcing their immune protection during the critical early life stages. Although IgG was not directly assessed in the current study, the observed increases in total protein, globulin, and IgA concentrations in both ewes and their lambs strongly suggest enhanced passive transfer of immunity. Previous studies have shown that total globulin and IgA are reliable correlates of colostral IgG content and neonatal immune status in lambs (Tóthová et al. 2023; Övet 2023; Farooq et al. 2024).

In addition to immunoglobulins, lamb antioxidant activity was improved, as evidenced by higher GPx activity and TAC at birth. This confirms that maternal antioxidant supplementation is effectively transferred to neonates, as Övet (2023) reported. Such enhancement of antioxidant defenses may be critical in counteracting oxidative stress in the immediate postnatal environment. While short-term benefits are evident, the long-term impacts of continuous maternal α-LA supplementation on lamb productivity, survival, and reproductive performance merit further research. Recent studies have shown that dietary α-LA improves growth performance, enhances antioxidant enzyme activity, and modulates immune function in sheep (Zhang et al. 2021; Yang et al. 2023). Therefore, these results emphasize that maternal α-LA supplementation enhances colostrum’s immunological and antioxidative quality, ultimately benefiting neonatal immune development.

### Udder characteristics and milk yield

Ewes supplemented with α-LA had larger udders compared to controls. Further research is needed to confirm the impact of α-LA on udder development, as this outcome is not yet documented in the literature.

Throughout the study, ewes treated with α-LA had higher milk production, albeit not significantly higher. The lack of statistical significance could be related to supplementation duration, dosage, or interactions with other dietary parameters that were not considered in the experimental design. To fully assess α-LA’s effectiveness as a milk production stimulant, greater sample sizes, varying dosages, and longer supplementation periods are necessary.

### Colostrum and milk quality

Supplementation of α-LA achieved significant benefits that enhance the quality standards of both colostrum and milk in ewes. The high fat and SNF content in whole milk and colostrum provides a superior nutritional quality that could enhance lamb growth and survival. Farooq et al. (2024) referred to the importance of saving quality colostrum to lambs in the first two weeks postpartum to preserve high survival rates. The newborn lamb’s energy is governed by increased fat in milk and colostrum.

The enhanced immunoglobulin content in our α-LA-supplemented ewes shows how α-LA increases immune functionality and antioxidative defenses. This result is consistent with Averós et al. (2022), who reported that α-LA-supplementation in ewes improved fatty acid compositions of colostrum with higher conjugated linoleic acid concentration, which sustains immune function. The enhanced immunoglobulin content of colostrum and milk helps better transfer passive immunity, which is vital for newborn lambs’ health (Övet 2023).

The colostrum of the α-LA group showed significant increasing trends in GPx and ALP activities. The rise in GPx activity appeared most obviously in colostrum because it keeps cells from oxidative damage, supporting maternal health and neonatal survival (Çolakoğlu et al. 2021). GPx in colostrum helps create a healthy gut microbiome, which is crucial for avoiding infections and promoting newborn nutrient absorption, as suggested by Sun et al. (2019). ALP also supports the maturation of the immune system, lowering the incidence of disease and enhancing general health gains in lambs (Wu et al. 2022). α-LA supplementation offers greater nutritional and immunological quality and attributes to whole milk and colostrum, which are expected to contribute to better lamb growth and survival. Future research will have to determine the impact of such improvements on lamb growth and flock productivity.

### Milk and colostrum hygiene

The absence of detectable microorganisms in the α-LA–treated group may be attributed to the improved immune and antioxidant status of treated ewes, as reflected by their higher TAC and immunoglobulin levels. Additionally, some pathogens, such as *Staphylococcus aureus*, exhibit intermittent shedding and may reside in udder tissues or supramammary lymph nodes, leading to temporary bacteriological clearance. These mechanisms, combined with enhanced immunity, likely contributed to the absence of bacterial growth in the α-LA group.

α-LA supplementation effectively reduced microbial contamination in colostrum and milk. The absence of *S. aureus*, coagulase-negative *staphylococci*, *E. coli*, *B. cereus*, and *Bacillus* spp. in the colostrum of supplemented ewes suggests that α-LA can enhance colostral antimicrobial efficiency by modulating immune responses and the microbiological balance of the mammary gland (Grayczyk and Alonzo 2019). This supplementation also helped preserve milk compositional hygiene, thereby improving lamb health and ensuring safer dairy products.

Furthermore, α-LA dietary supplementation minimized the occurrence of pathogenic bacteria, confirming its potential as a natural enhancer of milk quality in dairy ewes. Since mastitis is closely related to immune regulation (Chang et al. 2017), α-LA may exert its protective effect through modulation of nuclear factor-kappa B (NF-κB) signaling and the secretion of anti-inflammatory and proinflammatory cytokines (Li et al. 2015; Wu et al. 2022). α-LA has also been reported to show dose-dependent bacteriostatic effects against *Streptococcus* spp., *E. coli*, *S. aureus*, methicillin-resistant *S. aureus*, and *S. epidermidis* (Grayczyk and Alonzo 2019). Collectively, these outcomes emphasize α-LA as a promising natural additive for improving udder health and producing cleaner, higher-quality milk in dairy sheep.

### Growth performance of lambs

The current results show that lamb growth and weight development profited substantially through α-LA supplementation throughout the pre-weaning period. Throughout all the assessments (weeks 2, 4, and 6, lambing), weight recording revealed α-LA supplementation to have induced improved growth of lambs. Weighed weights revealed significant differences between α-LA-group lambs and controls at each observation time, and this pattern helped improve overall weight gain up to weaning age. Oxidative stress caused by pregnancy has been shown to produce adverse effects on fetal growth (Gao et al. 2025). By inhibiting oxidative stress, α-LA perhaps improved placental effectiveness, maternal hemodynamics, and fetal delivery of nutrients, resulting in higher birth weights (Barrientos et al. 2024). In addition, α-LA controls metabolic pathways, for example, the pentose phosphate pathway, producing NADPH, an essential cofactor in antioxidant defense (Yao et al. 2024). Such mechanisms might be responsible for promoting fetal and postnatal growth.

The current results are consistent with previous reports wherein α-LA improved feed consumption and antioxidant enzyme activity of ewes and lambs (Yang et al. 2023), and wherein Dorset × Pelibuey lambs fed α-LA exhibited improved weight gain and feed efficiency (Robles-Rodríguez et al. 2022). Nevertheless, caution is warranted when considering high doses, as they have been associated with reductions in volatile fatty acid concentrations, essential for rumen health and energy supply (Zhang et al. 2024). The present study proves that prepartum α-LA supplementation enhances lamb growth performance by improving maternal antioxidant capacity, placental efficiency, colostrum, and milk quality.

## Conclusion

Supplementing ewes with α-LA (600 mg/day) during the prepartum period enhanced the health and performance of ewes and lambs. It improved colostrum and milk composition and milk hygiene. Lambs of supplemented ewes had greater immunity and better growth performance. α-LA represents a promising management strategy in sheep production systems. Further studies should investigate dose-response relationships, the long-term effects of α-LA on milk yield and composition during complete lactation, and its role in mastitis control programs.

## Data Availability

The data sheets are available from the corresponding author if the editor requests them from the journal while publishing the current paper.
